# Improved Detection of Intestinal Helminth Infections with a Formalin Ethyl-Acetate-Based Concentration Technique Compared to a Crude Formalin Concentration Technique

**DOI:** 10.3390/tropicalmed6020051

**Published:** 2021-04-15

**Authors:** Tobias Brummaier, Laypaw Archasuksan, Dorn Watthanakulpanich, Daniel H. Paris, Jürg Utzinger, Rose McGready, Stephane Proux, François Nosten

**Affiliations:** 1 Shoklo Malaria Research Unit, Mahidol–Oxford Tropical Medicine Research Unit, Faculty of Tropical Medicine, Mahidol University, P.O. Box 46, 68/30 Bann Thung Road, Mae Sot 63100, Thailand; laypaw@shoklo-unit.com (L.A.); rose@shoklo-unit.com (R.M.); steph@shoklo-unit.com (S.P.); francois@tropmedres.ac (F.N.); 2 Swiss Tropical and Public Health Institute, P.O. Box, CH-4002 Basel, Switzerland; daniel.paris@swisstph.ch (D.H.P.); juerg.utzinger@swisstph.ch (J.U.); 3 University of Basel, P.O. Box, CH-4003 Basel, Switzerland; 4 Centre for Tropical Medicine and Global Health, Nuffield Department of Medicine, University of Oxford, Oxford OX3 7LG, UK; 5 Faculty of Tropical Medicine, Department of Helminthology, Mahidol University, 420/6 Ratchawithi Road, Ratchathewi, Bangkok 10400, Thailand; dorn.wat@mahidol.edu

**Keywords:** diagnosis, formalin ethyl-acetate concentration technique, small liver flukes, soil-transmitted helminths, stool concentration technique

## Abstract

Intestinal helminth infections are the most prevalent neglected tropical diseases, predominantly affecting rural and marginalised populations. The mainstay of diagnosis is the microscopic examination of faecal samples to detect parasites in the form of eggs, larvae and cysts. In an effort to improve the standard of care, the comparative accuracy in detecting helminth infections of the hitherto used formalin-based concentration method (FC) was compared to a previously developed formalin ethyl-acetate-based concentration technique (FECT), prior to the systematic deployment of the latter at a research and humanitarian unit operating on the Thailand–Myanmar border. A total of 693 faecal samples were available for the comparison of the two diagnostic methods. The FECT was superior in detecting hookworm, *Trichuris trichiura* and small liver flukes. Interestingly, there was no significant difference for *Ascaris lumbricoides*, possibly due to the high observed egg density. Despite the minor increase in material cost and the fact that the FECT is somewhat more time consuming, this method was implemented as the new routine technique.

## 1. Introduction

Helminths, which are widespread across various populations mostly in tropical and subtropical regions, are the most common infectious agents worldwide [1]. Contrary to their vast distribution, helminth infections were included in the list of neglected tropical diseases since the establishment of this denomination in 2005 and still retain a position in the most recent portfolio of neglected tropical diseases put forward by the World Health Organization (WHO) [2,3]. Their importance as a public health issue in some regions is underlined by the fact that about 1.5 billion people are estimated to be infected with soil-transmitted helminths (i.e., *Ascaris lumbricoides*, the two hookworm species *Ancylostoma duodenale* and *Necator americanus* and *Trichuris trichiura*), causing an estimated global burden of 2 million disability-adjusted life years (DALYs) [4,5]. Food-borne trematode infections are estimated to be liable for another 1.8 million DALYs [5]. Two species belonging to the food-borne trematodes (i.e., *Clonorchis sinensis* and *Opisthorchis viverrini*) have been listed as Group 1 carcinogens by the International Agency for Research on Cancer as they are risk factors for cholangiocarcinoma [6]. Accordingly, the need for an accurate diagnosis is evident. While tropical regions carry the highest burden of intestinal helminth infections, they mostly rely on microscopy as the sole diagnostic tool as molecular-based methods are usually not available because of financial and human resources constraints.

Shoklo Malaria Research Unit (SMRU), a field station of the Mahidol–Oxford University Research Unit, operates humanitarian clinics in such a tropical setting, serving a rural, migrant population alongside the Thailand–Myanmar border. Data from routine antenatal screening for intestinal parasitic infections in the pregnant population suggest a high burden of helminth infections [7,8]. The estimated prevalence in these reports was based on data derived from a formalin concentration (FC) stool examination method. Direct examination of wet mounts of samples preserved in formalin is a simple and inexpensive technique but lacks sensitivity when compared to other techniques [9]. Enhanced test accuracy does not only lead to a direct benefit on a patient level, it is also of significance to inform and determine public health policies, such as deworming strategies of vulnerable populations [10].

While the presence of helminth infections was known to humans thousands of years ago, the importance of examining faecal samples to detect intestinal helminth infections was first appreciated in the 19th century [11]. Different examination methods have been developed with the result of a gradual improvement of the diagnostic accuracy. A century ago, Telemann reported that the addition of ether prior to the filtering and centrifugation of suspended faecal material led to a marked improvement in the detection of helminth eggs in faecal samples [12]. This was mostly attributed to the fact that the solvent ether extracts fat and debris and that the lower specific gravity than the parasitic organisms, concentrates the latter in the sediment. Subsequently, Young and colleagues reported favourable results when replacing the highly flammable solvent ether with the less hazardous ethyl-acetate, a solvent with otherwise similar properties [9].

Seeking to improve services, the FC method was replaced by a formalin ethyl-acetate concentration technique (FECT) at SMRU. Here, we report the improved diagnostic comparative accuracy of the newly implemented technique with a focus on the detection of soil-transmitted helminths and food-borne trematodes, which motivated our decision to change routine diagnostics.

## 2. Materials and Methods

Fresh faecal samples were collected from individuals who would normally undergo testing (e.g., routine antenatal screening, or if clinically indicated and ordered by the attending physician). All faecal samples were collected in a small plastic container and immediately transferred to the onsite clinic laboratory, where the samples were partitioned for downstream processing by the hitherto used FC method and the FECT. The FC technique was applied following sample partitioning in the onsite laboratory, while the specimen designated for the FECT was kept at 4 °C and transferred to the central laboratory. If the specimens could not be examined on the same day of sample collection, the sample was stored overnight in a fridge at 4 °C.

### 2.1. FC Method

Stool samples (~500 mg) were mixed with 10 mL of 10% formalin solution and homogenized until all faecal material was suspended. Thereafter the tube was vigorously shaken to emulsify the faecal material. This emulsion was then filtered through a moulded strainer (Caplugs Evergreen FPC^®^ Fecal Parasite Concentrator; Rancho Dominguez, California, United States of America), whose 0.6 mm × 0.6 mm sieve opening size allow parasite eggs to pass though the strainer, while excess faecal debris was retained. After centrifugation (500 g for 2 min) in a conical centrifuge tube, the supernatant was discarded, and the remaining deposit was re-suspended in a 0.85% saline solution.

### 2.2. FECT

**Figure 1:**
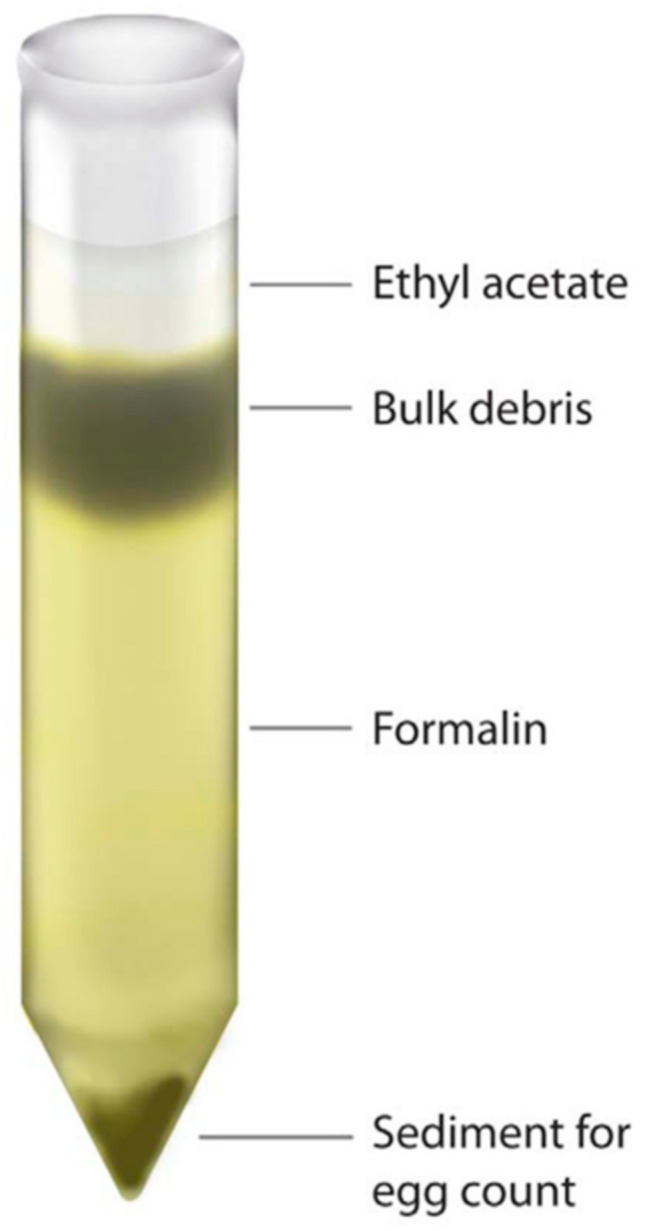
Layer of separation after the addition of ethyl-acetate and centrifugation. Reprinted from Xu et al. (2012) [13].

Faecal material (~500 mg) was added to 10 ml of clean water and mixed. Then, the specimen was vigorously shaken and filtered through a moulded strainer, identical to the one mentioned above. After the suspension was centrifuged (500 g for 5 min), the supernatant was discarded, and the sediment was re-suspended with 10 ml of a 10% formalin solution. Thereafter, 4 mL of ethyl-acetate was added, the solution was vigorously shaken for 30 s and then centrifuged at 500 g for 5 min in a conical centrifuge tube. The plug of debris that formed at the top of the tube was freed and the top layers of the supernatant containing ethyl-acetate, debris and formalin were decanted (Figure 1). Remaining debris were removed from the side of the tube with a cotton tipped applicator. Lastly, the concentrated sediment for the egg specimen was resuspended with 0.85% saline solution.

### 2.3. Microscopic Examination

For both diagnostic methods, wet mounts with 1–2 drops were taken from the tip of the conical tube of each solution and prepared on microscope slides, and each slide was examined by two experienced microscopists following a systematic reading procedure. Examiners reading the slides prepared with the FC method were blinded to the results from examiners using FECT and vice versa. In a positive sample, a semi-quantitative approach to estimate the intensity of infection was adapted. The intensity of infection was categorized into rare, low, medium and high (1, 2–3, 4–10 and >10 eggs per slide, respectively).

### 2.4. Statistical Analysis

R (version 3.5.1) was used for analysis. Agreement, calculated by the *irr* package, version 0.84.1, was expressed by the crude proportion of agreement and kappa (κ) statistics [14,15]. To estimate the comparative diagnostic accuracy, both methods were combined in a composite reference standard, following an *any positive* rule [16]. The rationale for this approach is based on the fact that false positive results in a visual-empirical test are presumably rare [17]. Sensitivity, specificity, positive predictive value (PPV) and negative predictive value (NPV) were calculated with the *epiR* package, version 0.9–99 [18]. Intensity of infection was compared by a Mann–Whitney U test only for the samples in which both methods agreed on positivity.

### 2.5. Ethics Statement

As the primary objective was the improvement of the standard of care, this comparison was considered a review of services and the Oxford Tropical Research Ethic Committee granted a waiver from full ethical board review.

## 3. Results

Overall, 693 faecal samples collected between February 2018 and July 2018 were available for comparison with the two diagnostic methods. The majority of samples originated from routine screening procedures (e.g., routine antenatal screening) of asymptomatic individuals, while some were taken from symptomatic individuals in the clinical diagnostic process. Taken together, 47.6% (330/693) of all samples contained at least one human pathogenic helminth species. In 67.0% (221/330) of positive samples, a mono-infection with one helminth species was reported, 27.3% (90/330) contained two, 4.5% (15/330) three and 1.2% (4/330) four different species. Soil-transmitted helminths were accountable for the vast majority of positivity: hookworm (i.e., *A. duodenale* and *N. americanus*), with a cumulative prevalence of 23.9% (166/693), followed by *T. trichiura* (17.3%, 120/693) and *A. lumbricoides* (9.1%, 63/693). Small liver flukes (*C. sinensis* and *O. viverrini*; as these two species are difficult to distinguish, they were combined in a single group) were detected in 13.7% (95/693), while other helminth infections were negligible (Table S1).

The FECT was superior to the FC method in detecting hookworm (145 vs. 89, *p* < 0.001), *T. trichiura* (109 vs. 53, *p* < 0.001) and small liver flukes (85 vs. 39, *p* < 0.001), while no difference was seen for *A. lumbricoides* (50 vs. 57, *p* = 0.546). Test performances for selected helminth species are summarized in Table 1 with more details are provided in Table S2.

**Figure 2:**
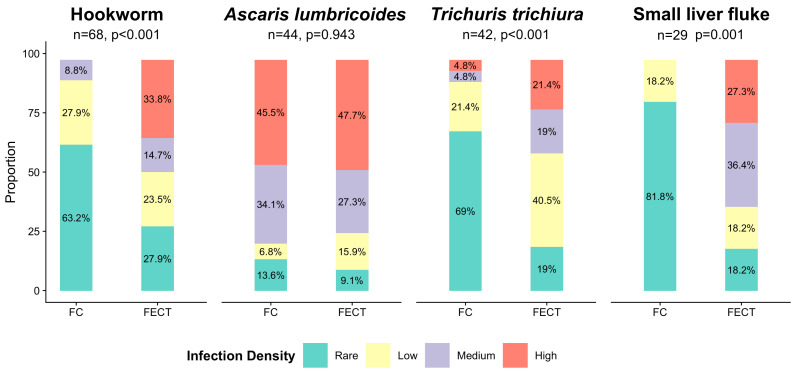
Helminth species-specific infection intensity compared between samples on which both methods agreed on positivity.

Infection intensities were compared for hookworm, *A. lumbricoides*, *T. trichiura* and small liver flukes if both methods agreed on positivity. In line with test sensitivities, higher egg densities were detected by FECT for hookworm (*p* < 0.001), *T. trichiura* (*p* < 0.001) and small liver flukes (*p* = 0.001), while no difference was seen for *A. lumbricoides* (*p* = 0.943) (Figure 2).

**Scheme for Kappa (**κ**)**. Fair agreement (κ = 0.21–0.40), moderate agreement (κ = 0.41–0.60), substantial agreement (κ = 0.61–0.80) and almost perfect agreement (κ = 0.81–1.00) [14].

## 4. Discussion

FECT was superior in detecting helminth infections when compared to the FC method in 693 individuals who consulted a health facility on at Thailand–Myanmar border. When combined into a composite reference standard, positive samples processed by FECT were correctly identified in 90.6% (299/330) compared to 61.2% (202/330) when the FC method was used. These findings were anticipated and are supported by and in agreement with previous reports [9]. The superiority of FECT was evident for hookworm, *T. trichiura* and small liver fluke but not for *A. lumbricoides*. The deviation in sensitivity for the detection of *A. lumbricoides* eggs from the overall trend observed for other helminth species and the similar distribution of infection intensity for this particular species is not entirely clear. Increased detection rates are expected for any microscopy-based examination in the event of high egg abundance. The high egg density (medium or high in more than 75% for both methods) and the substantial agreement between both methods (κ = 0.81) are indicators of the high intensity of infection for *A. lumbricoides*, which might have led to a comparable performance of both techniques for this particular helminth species.

The FECT entailed a 25% increase in material cost when compared to the FC method and it is somewhat more time consuming. However, the significance of continuous review and improvement of diagnostic standards lies in the direct patient benefit via increased test sensitivities and the necessity to provide accurate data to public health stakeholders to enable an evidence-based decision-making process regarding public health interventions. Usually, intestinal helminth infections are of low priority as they are overshadowed by other communicable diseases that are associated with higher morbidity and mortality (e.g., microscopic examination of blood films for *Plasmodium*, the causative agent of malaria). In our unit, a substantial reduction in malaria incidence rates, freed resources that were re-directed to implement the FECT for helminth diagnosis.

The strength of this comparison is the fact that the same faecal samples were used to investigate the performance of the respective technique and that these samples were derived from actual clinical work. The latter ensures that the findings are directly applicable to the future use of the newly implemented method. While an effort was made to limit the effect of inter-rater variability by reading slides in duplicate and by following a standard operating procedure, it remains a noteworthy limitation of coproscopic examinations [19]. In addition, intra-specimen variability may have contributed to differing results [20]. Kato–Katz thick smears allow a more precise estimation of infection intensity, are commonly employed for the diagnosis of intestinal schistosomiasis and soil-transmitted helminths worldwide and are recommended by WHO [21]. Ideally, Kato–Katz thick smear data would have been included in this comparison, hence this can be seen as a limitation of the study. However, Kato–Katz thick smears have been linked to a lower sensitivity in detecting hookworm eggs, in particular if there are delays between sample production, the preparation of Kato–Katz thick smears in the laboratory and slide reading under a microscope by laboratory technicians [21,22]. While hookworms are prevalent in the region where SMRU operates, schistosomiasis is absent [7,23]. Hence, FECT was chosen over Kato–Katz as the routine stool examination technique in the clinics.

FECT allows the simultaneous diagnosis of intestinal protozoal infections. The concurrent diagnosis of intestinal protozoal infections relies on immediate direct exams of wet mounts in some instances (e.g., *Giardia intestinalis*). As direct wet mount exams were not routinely done prior to sample processing, findings for intestinal protozoal infections are not reported here.

Data derived from routine screening and surveillance, based on the newly deployed, more precise FECT method, will enable a better informed decision-making process as to whether current procedures (e.g., WHO recommendation to provide routine preventive chemotherapy to all pregnant women attending antenatal care) need to be reviewed and/or adopted [10]. Moreover, the increased accuracy of diagnostic tools may reveal knowledge gaps in the local prevalence and distribution of intestinal parasitic infections and provide improved information about re-infection rates as well as the efficacy of the currently provided anthelminthic treatment using albendazole or mebendazole, and thus indicate directions of further translational research. On a wider scope, accurate and reproducible diagnostics by using tools that are currently available in resource-limited settings are needed to inform the WHO 2021–2030 Roadmap for Neglected Tropical Diseases [24]. In addition to diagnostics, these infectious diseases of poverty confront health care systems with the challenge to mobilize cross-sectorial action and link with municipalities, local governments and community-based organizations for basic water supply, adequate sanitary infrastructure and improved hygiene and housing [25].

## 5. Conclusions

In conclusion, the adaption of FECT as the new standard diagnostic method will increase our understanding of intestinal helminthiasis in this population. The results reported here may also act as a stimulus to encourage other health care facilities in similar settings to review their standard practices.

## Figures and Tables

**Table 1:** Cross-tabulation of test performance for the diagnosis of hookworm (i.e., *Ancylostoma duodenale* and *Necator americanus*), *Trichuris trichiura*, *Ascaris lumbricoides* and small liver flukes.

Hookworm (*p* < 0.001 ^⧧^)					*Trichuris trichiura* (*p* < 0.001 ^⧧^)				
		FECT (sensitivity 87.3%)					FECT (sensitivity 90.8%)		
		−	+	Total			−	+	Total
FC method (sensitivity 53.6%)	−	527	77	604	FC method (sensitivity 44.1%)	−	573	67	640
	+	21	68	89		+	11	42	53
	Total	548	145	693		Total	584	109	693
Kappa = 0.50, PoA = 85.9%					Kappa = 0.46, PoA = 88.7%				
***Ascaris lumbricoides*** **(*p* = 0.546** **^⧧^** **)**					**Small liver fluke (*p* < 0.001** **^⧧^** **)**				
		FECT (sensitivity 79.4%)					FECT (sensitivity 89.5%)		
		−	+	Total			−	+	Total
FC method (sensitivity 90.5%)	−	630	6	636	FC method (sensitivity 41.1%)	−	598	56	654
	+	13	44	57		+	10	29	39
	Total	643	50	693		Total	608	85	693
Kappa = 0.81, PoA = 97.3%					Kappa = 0.30, PoA = 93.5%				

^⧧^ Comparison of the detection rate between the FECT and FC method (χ^2^ test). FC: formalin concentration; FECT: formalin ethyl-acetate concentration technique; PoA: proportion of agreement.

## Data Availability

Data cannot be shared publicly as this is a population of undocumented migrants. Data are available from the Mahidol–Oxford Research Unit Institutional Data Access Committee (contact Rita Chanviriyavuth, email: rita@tropmedres.ac) for researchers who meet the criteria for access to confidential data.

## Supplementary Materials

The following are available online at https://www.mdpi.com/article/10.3390/tropicalmed6020051/s1, Table S1. Number of stool samples positive for helminth infections; Table S2. Comparative diagnostic accuracy of the FECT and FC methods for different helminth species.
