# Supplementary material for: Improved Detection of Intestinal Helminth Infections with a Formalin Ethyl-Acetate-Based Concentration Technique Compared to a Crude Formalin Concentration Technique
Source: Trop Med Infect Dis. 2021 Apr 15;6(2):51. doi: 10.3390/tropicalmed6020051 (PMC8167623; doi:10.3390/tropicalmed6020051)
Supplement: Supplementary file 1 [file tropicalmed-06-00051-s001.zip › tropicalmed-1178600-supplementary.pdf]

Table S1. Number of stool samples positive for helminth infections.

	Combined results		FECT		FC		PoA	Kappa	p-value†
	N (%)	95% CI	N (%)	95% CI	N (%)	95% CI			
Overall	330 (47.6)	43.9–51.3	299 (43.2)	39.5–46.7	202 (29.2)	25.9–32.6	77.1	0.513	< 0.001‡
Hookworm	166 (23.9)	20.9–27.3	145 (20.9)	18.1–24.1	89 (12.8)	10.6–15.5	85.9	0.502	< 0.001‡
Tricuris trichiura	120 (17.3)	14.7–20.3	109 (15.7)	13.2–18.6	53 (7.7)	5.9–9.9	88.7	0.463	<0.001‡
Asacaris lumbricoides	63 (9.1)	7.2–11.5	50 (7.2)	5.5–9.4	57 (8.2)	6.4–10.5	97.3	0.808	0.546‡
Small liver fluke	95 (13.7)	11.4–16.5	85 (12.3)	10.0–14.9	39 (5.6)	4.1–7.6	93.5	0.295	<0.001‡
Strongyloides stercoralis larvae	7 (1.0)	0.49–2.1	6 (0.87)	0.4–1.88	1 (0.14)	0–0.81	99	-0.003	0.124*
Trichostrongylus spp.	4 (0.58)	0.22–1.5	2 (0.29)	0.08–1.1	2 (0.29)	0.08–1.1	99.4	-0.003	1*
Hymenolepis nana	3 (0.43)	0.15–1.3	3 (0.43)	0.15–1.3	1 (0.14)	0.03–0.81	99.7	0.499	0.625*
Enterobius vermicularis	3 (0.43)	0.15–1.3	3 (0.43)	0.15–1.3	0 (0)	0–0.55	99.6	0	0.250*
Taenia spp.	1 (0.14)	0.03–0.81	1 (0.14)	0.03–0.81	0 (0)	0–0.55	99	0	1*

Data shown as n (%) for combined results, FECT and FC.

† Comparison of the FECT and FC methods.

‡ Chi-square test

* Fisher exact test

Kappa: no agreement (κ < 0), slight agreement (κ = 0–0.20), fair agreement (κ = 0.21–0.40), moderate agreement (κ = 0.41–0.60), substantial agreement (κ = 0.61–0.80) and almost perfect agreement (κ = 0.81–1.00)

Abbreviations: FC, formalin concentration technique; FECT, formalin-ethyl-acetate concentration technique; PoA, proportion of agreement.

Table S2. Comparative diagnostic accuracy of the FECT and FC methods for different helminth species.

		Sensitivity*	Specificity*	PPV*	NPV*
Total	FECT	0.91 (0.87–0.94)	1.00 (0.99–1.00)	1.00 (0.99–1.00)	0.92 (0.89–0.95)
	FC	0.61 (0.56–0.66)	1.00 (0.99–1.00)	1.00 (0.98–1.00)	0.74 (0.70–0.78)
Hookworm	FECT	0.87 (0.81–0.92)	1.00 (0.99–1.00)	1.00 (0.97–1.00)	0.96 (0.94–0.98)
	FC	0.54 (0.46–0.61)	1.00 (0.99–1.00)	1.00 (0.96–1.00)	0.87 (0.84–0.90)
Trichuris trichiura	FECT	0.91 (0.84–0.95)	1.00 (0.99–1.00)	1.00 (0.97–1.00)	0.98 (0.97–0.99)
	FC	0.44 (0.35–0.54)	1.00 (0.99–1.00)	1.00 (0.93–1.00)	0.90 (0.87–0.92)
Ascaris lumbricoides	FECT	0.79 (0.67–0.89)	1.00 (0.99–1.00)	1.00 (0.93–1.00)	0.98 (0.97–0.99)
	FC	0.90 (0.80–0.96)	1.00 (0.99–1.00)	1.00 (0.94–1.00)	0.99 (0.98–1.00)
Small liver fluke	FECT	0.89 (0.81–0.95)	1.00 (0.99–1.00)	1.00 (0.96–1.00)	0.98 (0.97–0.99)
	FC	0.41 (0.31–0.52)	1.00 (0.99–1.00)	1.00 (0.91–1.00)	0.91 (0.89–0.93)
Strongyloides stercoralis	FECT	0.86 (0.42–1.00)	1.00 (0.99–1.00)	1.00 (0.54–1.00)	1.00 (0.99–1.00)
larvae	FC	0.14 (0.00–0.58)	1.00 (0.99–1.00)	1.00 (0.03–1.00)	0.99 (0.98–1.00)
Trichostrongylus spp.	FECT	0.50 (0.07–0.93)	1.00 (0.99–1.00)	1.00 (0.16–1.00)	1.00 (0.99–1.00)
	FC	0.50 (0.07–0.93)	1.00 (0.99–1.00)	1.00 (0.16–1.00)	1.00 (0.99–1.00)
Hymenolepis nana	FECT	1.00 (0.29–1.00)	1.00 (0.99–1.00)	1.00 (0.29–1.00)	1.00 (0.99–1.00)
	FC	0.33 (0.01–0.91)	1.00 (0.99–1.00)	1.00 (0.03–1.00)	1.00 (0.99–1.00)
Enterobius vermicularis	FECT	1.00 (0.03–1.00)	1.00 (0.99–1.00)	1.00 (0.03–1.00)	1.00 (0.99–1.00)
	FC	0.00 (0.00–0.71)	1.00 (0.99–1.00)	NA	NA
Taenia spp.	FECT	1.00 (0.03–1.00)	1.00 (0.99–1.00)	1.00 (0.03–1.00)	1.00 (0.99–1.00)
	FC	0 (0.00–0.98)	1.00 (0.99–1.00)	NA	NA

Data shown as % and 95% confidence interval.

Abbreviations: NPV, negative predictive value; PPV, positive predictive value.
